# Epitranscriptomics and epiproteomics in cancer drug resistance: therapeutic implications

**DOI:** 10.1038/s41392-020-00300-w

**Published:** 2020-09-08

**Authors:** Huibin Song, Dongcheng Liu, Shaowei Dong, Leli Zeng, Zhuoxun Wu, Pan Zhao, Litu Zhang, Zhe-Sheng Chen, Chang Zou

**Affiliations:** 1 Shenzhen People’s Hospital (The Second Clinical Medical College, Jinan University; The First Affiliated Hospital, Southern University of Science and Technology), Shenzhen, 518001 Guangdong China; 2 College of Pharmacy and Health Sciences, St. John’s University, Queens, 11439 New York, USA; 3 Tomas Lindahl Nobel Laureate Laboratory, Research Centre, The Seventh Affiliated Hospital, Sun Yat-sen University, Shenzhen, 518107 Guangdong China; 4 Department of Research, Affiliated Tumor Hospital of Guangxi Medical University, Nanning, 530021 Guangxi China; 5 Shenzhen Public Service Platform on Tumor Precision Medicine and Molecular Diagnosis, Shenzhen, 518001 Guangdong China

**Keywords:** Gene therapy, Drug development

## Abstract

Drug resistance is a major hurdle in cancer treatment and a key cause of poor prognosis. Epitranscriptomics and epiproteomics are crucial in cell proliferation, migration, invasion, and epithelial–mesenchymal transition. In recent years, epitranscriptomic and epiproteomic modification has been investigated on their roles in overcoming drug resistance. In this review article, we summarized the recent progress in overcoming cancer drug resistance in three novel aspects: (i) mRNA modification, which includes alternative splicing, A-to-I modification and mRNA methylation; (ii) noncoding RNAs modification, which involves miRNAs, lncRNAs, and circRNAs; and (iii) posttranslational modification on molecules encompasses drug inactivation/efflux, drug target modifications, DNA damage repair, cell death resistance, EMT, and metastasis. In addition, we discussed the therapeutic implications of targeting some classical chemotherapeutic drugs such as cisplatin, 5-fluorouridine, and gefitinib via these modifications. Taken together, this review highlights the importance of epitranscriptomic and epiproteomic modification in cancer drug resistance and provides new insights on potential therapeutic targets to reverse cancer drug resistance.

## Introduction

### Drug resistance in cancer treatment

Cancer remains the leading cause of incidence and mortality worldwide.^1,2^ The development of cancer is a complex process with significant biological characteristics, such as abnormal cell proliferation and differentiation, a high degree of molecular heterogeneity and epithelial–mesenchymal transition (EMT).^3^ Because most cancers have progressed to the middle or late stages when diagnosed, molecular targeted drug therapy and chemotherapy are the main treatment options.^4^ The most common therapeutic drugs include cisplatin, sorafenib, oxaliplatin, 5-fluorouracil, and epidermal growth factor receptor-tyrosine kinase inhibitors (EGFR-TKIs).^5^ However, long-term therapies usually lead to acquired drug resistance and poor prognosis. The main underlying mechanisms of drug resistance include: (1) drug efflux and alterations in drug metabolism; (2) alterations of drug targets; (3) DNA damage repair (DDR); (4) deregulation of apoptosis and autophagy; (5) resistance-promoting adaptive responses; (6) alterations in tumor microenvironment; and (7) epigenetic changes.^6,7^

Epigenetics refers to a “heritable” phenomenon in which the phenotype changes are independent of DNA sequence. Epitranscriptomics, also called “RNA epigenetics,” is a branch of epigenetics and refers RNA editing and noncoding RNA regulations. Epitranscriptomics plays essential roles in alternative splicing, nuclear export, transcript stability, and translation of RNAs.^8,9^ Epiproteomics is the posttranslational modifications (PTMs) that involve histone acetylation, SUMOylation, phosphorylation, and ubiquitination.^10–12^ The PTMs might regulate various biological processes via modulating chromosomal structures or regulating the binding of chromatin.^13^ Recent studies in RNA and protein modifications mainly focus on evaluating drug response to screening drugs suitable for individual patients or as the molecular targets to pioneer new ways of cancer treatment.^14–17^ In this review, we will discuss the role of posttranscriptional and PTM in cancer drug resistance and therapeutic targets.

### mRNA modification in cancer drug resistance

**Fig. 1:**
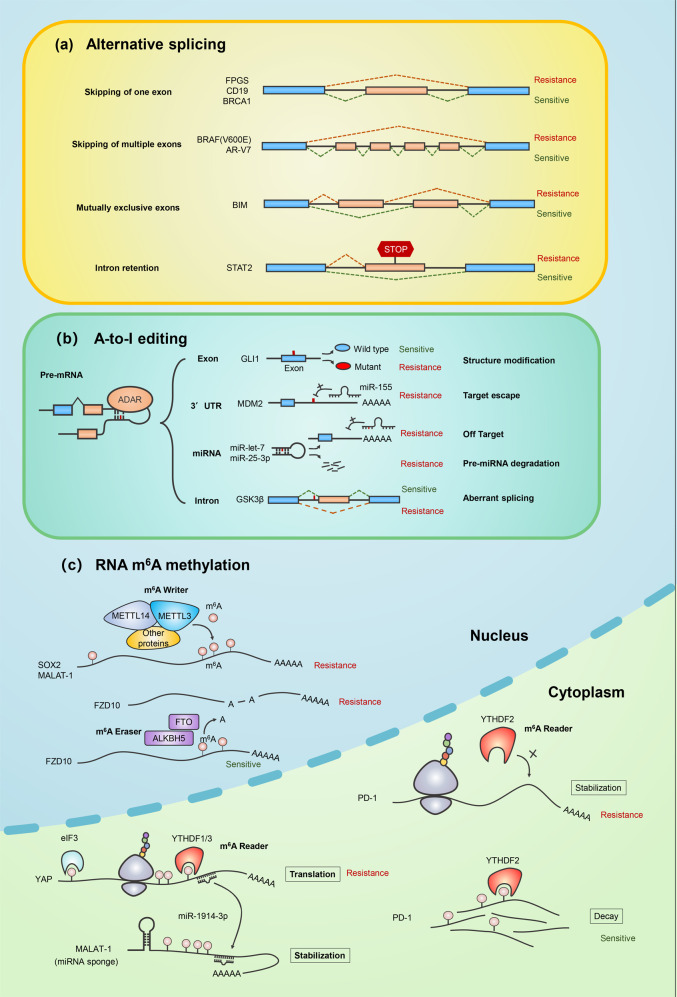
mRNA modification in cancer drug resistance. **a** Schematic representation of examples of alternative splicing patterns causing cancer drug resistance, including skipping of one exon, skipping of multiple exons, mutually exclusive exons, and exon inclusion. **b** Schematic representation of A-to-I RNA editing mediated drug-resistance-related functional consequences including structure modification of targeted protein, target escape from silencing of miRNA, off-target effects of miRNA, pre-miRNA degradation, aberrant splicing of targeted mRNA. **c** Schematic representation of m^6^A modification network in targeted genes causing cancer drug resistance. In the nucleus, m^6^A is deposited in nascent pre-mRNA by a “writer” multiprotein complex (i.e., METTL3, METTL14, and other related protein) and removed by “eraser” demethylases (i.e., FTO and ALKBH5). In the cytoplasm, the m^6^A modifications are recognized by “reader” proteins, resulting in stabilization or decay or enhanced translation. Specific examples of each mRNA modification event discussed in the text are shown

In this section, we focus on the mechanism of cancer drug resistance in three different types of RNA modifications: alternative splicing, adenosine-to-inosine (A-to-I) modification, and mRNA methylation (Fig. 1 and Table 1).

**Table 1:** A summary of mRNA modification associated cancer drug resistance

Cancer type	Target gene	RNA modification	Drug/ molecule	Molecular mechanisms	Reference
Skipping of one exon during splicing					
Leukemia	FPGS	Aberrant splicing	Methotrexate	Aberrant splicing induces skipping of exon 12 and generates a nonfunctional FPGS enzyme resulting in loss of antifolate retention and folate antagonist methotrexate resistance	^23^
B-ALL	CD19	Aberrant splicing	CART-19	Skipping of Exon 2 in CD19 allows expression of the N-terminally truncated CD19 variant causes failure of apoptosis triggered by CART-19 in B-ALL cells	^24^
Breast cancer, ovary cancer cells	BRCA1	Aberrant splicing	Cisplatin PARP inhibitors	Cells harboring BRCA1 splice variant lacking the majority of exon 11 promotes partial PARPi and cisplatin resistance relative to full-length BRCA1	^26^
Skipping of multiple exons during splicing					
Melanoma	BRAF V600E	Aberrant splicing	Vemurafenib BRAF inhibitor	BRAF3-9 (∆exons 4–8 or/and BRAF2–6 ∆ exons 3–5) which are eliminated of RAS-binding domain resulting to drug resistance	^27,28^
Prostate cancer	AR-V7	Aberrant splicing	Enzalutamide Abiraterone	AR-V7 (exon 4–8) lacking the ligand-binding domain are constitutively active that cause tumors resistant to androgen-targeted therapies	^29^
Mutually exclusive exons by aberrant splicing					
CML NSCLC	BIM	Aberrant splicing	TKIs	A polymorphism switched BIM splicing from exon 3 to exon 4 would result in deletion of pro-apoptotic BH3 domain and conferring intrinsic TKI resistance	^30^
Intron retention by aberrant splicing					
Burkitt lymphoma	STAT2	Aberrant splicing	IFN, camptothecin, staurosporine, doxorubicin	Splice variant of STAT2 contains intron 19 introducing a premature stop codon, leading to resistance drug	^31^
A-to-I editing in coding gene					
Myeloma	GLI1	A-to-I editing	Immunotherapy	ADAR1 causes an R701G mutation in GLI1, stabilizes GLI1 transcriptional activity, and results in promotion of immunomodulatory drug resistance	^38^
A-to-I editing in microRNA					
Leukemia	miR-let-7	A-to-I editing		ADAR1-mediated A-to-I editing impairs let-7 biogenesis leading to LSC self-renewal	^42^
Breast cancer	miR-25-3p	A-to-I editing	Methotrexate	ADAR1 induces the expression of DHFR and resistance to methotrexate by editing the miR-25-3p, which is the regulator of 3′UTR of DHFR	^43^
A-to-I editing in 3’ UTR					
CML	MDM2	A-to-I editing	Chemotherapy	ADAR1 modifies the 3′UTR region of MDM2 to prevent the binding and downregulation of miR-155 leading to enhancing the malignant reprogramming of progenitors into dormant leukemia stem cells	^44^
A-to-I editing in intron					
Leukemia	GSK3β	A-to-I editing	TKIs	A-to-I editing induced mis-splicing of GSK3β in LSC resulting in enhanced β-catenin expression, which is responsible for therapeutic resistance	^45^
m^6^A Writer regulation					
Glioma	SOX2	m^6^A methylation	Radiotherapy	METTL3 interacts with the 3′UTR of SOX2 mRNA leading to methylation and stabilization of mRNA of SOX2 in glioma stem-like cells	^63^
Lung cancer	EGFR	m^6^A methylation	TKIs	METTL3 shuttles from nuclear to cytoplasm and interacts with ribosomes resulting in promotion of EGFR mRNA translation	^64^
m^6^A Reader regulation					
AML	TNFRSF2	m^6^A methylation	TNF-induced apoptosis	YTHDF2 induces LSC development and propagation via decreasing the half-life of TNFRSF2	^65^
m^6^A Eraser regulation					
Ovarian cancer	FZD10	m^6^A methylation	PARP inhibitor	Deletion of FTO and ALKBH5 stabilizes FZD10 mRNA via enhancement of m^6^A modification, leading to overcome PARP inhibitor resistance	^66^
Regulation by multiple m^6^A regulators					
NSCLC	YAP	m^6^A methylation	Castration	METTL3 prevent the degradation of YAP by increasing LncRNA MALAT1, which is the sponge of YAP downregulator miRNA-1914-3p. METTL3 promotes YAP mRNA translation by recruiting YTHDF1/3 and eIF3b to the translation initiation complex machinery	^68^
Melanoma	PD-1	m^6^A methylation	PD-1 antibody	Overexpression of FTO decreases m^6^A methylation in PD-1 mRNA, leading to PD-1 mRNA decay through YTHDF2 regulation	^69^

*ADAR1* RNA-specific adenosine deaminase 1, *ALKBH5* AlkB homolog 5, RNA demethylase, *AML* acute myeloid leukemia, *AR-V7* androgen-receptor splice variant 7, *B-ALL* B-acute lymphoblastic leukemia, *BH3* Bcl-2 homology regions 3, *BIM (BCL-2-like 11)* bcl-2-interacting mediator of cell death, *BRAF* B-Raf proto-oncogene, serine/threonine kinase, *BRCA1* breast cancer genes 1, *CART-19* chimeric antigen receptor-modified T cells targeting the CD19 antigen, *CML* chronic myelogenous leukemia, *DHFR* dihydrofolate reductase, *EGFR* epidermal growth factor receptor, *ER* endoplasmic reticulum, *FPGS* folylpolyglutamate synthase, *FTO* fat mass and obesity-associated protein, *FZD10* frizzled class receptor 10, *GLI1* glioma-associated oncogene homolog 1, *GSK3 β* glycogen synthase kinase-3 beta, LSC leukemic stem cell, *IFN* interferon, *MDM2* mouse double minute 2, *METTL3* methyltransferase-like 3, *NCOR2* nuclear receptor corepressor 2, *NSCLC* non-small-cell lung cancer, *PARP* poly (ADP-ribose) polymerase 1, *PD-1* programmed cell death protein 1, *SOX2* SRY-Box transcription factor 2, *STAT2* signal transducer and activator of transcription 2, *TKI* tyrosine kinase inhibitor, *TNF* tumor necrosis factor, *TNFRSF2* tumor necrosis factor receptor superfamily member 2, *UTR* untranslated region, *YAP* yes associated protein, *YTHDF2* YTH N6-methyladenosine RNA binding protein 2

#### Alternative splicing and cancer drug resistance

Alternative splicing is a process by which introns are differentially removed from a single precursor mRNA (pre-mRNA) to generate multiple mature mRNA products.^18,19^ More than 95% of human genes are transcribed into pre-mRNAs that undergo alternative splicing.^20–22^ Since alternative splicing represents a frequent mechanism underlying the expansion of transcriptomes and proteomes in higher eukaryotes, it plays numerous critical roles in both normal and disease processes. Global analysis has revealed at least 15,000 cancer specific splice variants in 27 types of cancers,^20^ indicating that alternative splicing is a significant mechanism contributing to the progression of cancer development, including cell proliferation, apoptosis, invasion, metastasis, angiogenesis, and drug resistance.^21^ Thus, it is becoming increasingly clear that alternative splicing regulates many of the biological and pathological processes. Therefore, alternative splicing could be potential targets for the development of new cancer therapeutics.^22^ To illustrate the alternative splicing patterns and programs that cancer cells apply to gain drug resistance, we describe below an exemplary set of functionally important alternative splicing events.

Exon skipping is one of the most important alternative splicing processes in drug-resistant cancer cells. In leukemia, the enzyme folylpolyglutamate synthetase (FPGS) is responsible for the intracellular retention of folates and antifolates by polyglutamylation. Aberrant splicing of FPGS induced skipping of exon 12 and generated a nonfunctional FPGS enzyme, which leads to reduced retention of antifolates and causes cancer cells resistant to folate antagonist methotrexate.^23^ In patients with B-cell malignancies treated with adoptive T cells expressed chimeric antigen receptors against CD19 (CART-19), the expression of alternatively spliced CD19 isoform lacking exon 2 caused failure of initiation of CART-19-mediated cancer cell death.^24^ TGF-β-activated kinase 1 (TAK1) promoted TGF-β-induced apoptosis in response to TGF-β activation. However, TAK1 variable exon 12 exerted opposite function that constitutively supported TGF-β-induced EMT and activated nuclear factor-kappa B (NF-κB) signaling pathway, eventually causing chemotherapeutic resistance.^25^ Breast and ovary cancer cells can overcome deleterious germline mutations in BRCA1 (the gene encoding breast cancer type-1 susceptibility protein) by alternative splicing. Among the splicing products, BRCA1-Δ11q retains residual activity, triggering resistance to cisplatin and poly ADP-ribose polymerase (PARP) inhibitors.^26^

In order to escape from drug mediated apoptosis, targeted genes would undergo multiple exons skipping to delete the specific domains targeted by cancer drugs. BRAF is an oncogene belonging to RAS/MAPK signaling pathway, which controls several important cellular functions including proliferation and migration. About 90% of melanomas harbor BRAF V600E mutation, which leads to the constitutive activation of RAS/MAPK signaling pathway and malignant cell proliferation. Vemurafenib is a potent RAF kinase inhibitor with remarkable clinical activity against BRAF (V600E) melanoma. However, patients rapidly develop resistance to vemurafenib treatment. Mechanistically, patients harboring isoform BRAF3-9 (Δ exons 4–8) or BRAF2-6 (Δ exons 3–5) that could eliminate RAS-binding domains often develop drug resistance.^27,28^ Advanced prostate cancer is commonly treated with drugs that inhibit androgen biosynthesis or antagonize the interaction between androgen and androgen receptor (AR). AR splice variant 7, which lacked the ligand-binding domain (exon 4–8), was constitutively resistant to androgen-targeted therapies.^29^

Mutually exclusive exons represent a rare subtype of RNA splicing. However, once it occurs, the cells harboring the spliced product with drug-resistant function would be evolutionally selected and accumulated resulting in cancer drug resistance. B-cell CLL/lymphoma 2 (BCL-2)-like 11 (BIM), a pro-apoptotic member of the BCL-2 family, is required for TKIs to induce apoptosis in kinase-driven cancers. A polymorphism switched BIM splicing from exon 3 to exon 4 would result in deletion of pro-apoptotic BCL-2-homology domain 3 (BH3) and confer intrinsic TKI resistance in both CML and EGFR NSCLC cells. Patients with this mutant protein had a poorer response to tyrosine kinase inhibitors than individuals without the polymorphism.^30^ In addition to exon removal and switching, intron retention is another mechanism that cancer cells applied for drug resistance. Interferon (IFN) treatment is effective in hematological malignancies through mediating cell apoptosis. Signal transducer and activator of transcription 2 (STAT2) is a transcription factor that contributes to the activation of IFN responsive genes. However, cancer cells frequently develop a new splice variant of STAT2 that contains intron 19 and a premature stop codon, leading to resistance to apoptosis induced by IFN and a number of chemotherapeutic agents (camptothecin, staurosporine, and doxorubicin (DOX)).^31^

#### A-to-I modification and cancer drug resistance

In eukaryotes, A-to-I editing in double-stranded RNA is one of the most prevalent RNA modifications. This process involves hydrolytic deamination of adenosine, catalyzed by the adenosine deaminase acting on RNA (ADAR) family members (ADAR1, ADAR2, and ADAR3).^32^ The newly generated inosine base is interpreted by the ribosome as guanosine, and this event could occur in protein-coding region during mRNA translation, leading to altered protein products,^33,34^ noncoding regions, such as introns, 5′ and 3′ untranslated regions (UTRs), and repetitive sequences, such as human Alu elements.^35^ Deregulation of ADAR1 has emerged as a dominant driver of cancer progression and therapeutic resistance.^36^ The transition from pre-malignant progenitor to therapy resistant cancer stem cell (CSC) is often accompanied by aberrant ADAR1 activation.^37^ The most common mechanism involved in A-to-I editing induced drug resistance during therapeutic treatment is protein mutation. In multiple myeloma, ADAR1 enhanced Alu-dependent editing and transcriptional activity of GLI1, a Hedgehog (Hh) signaling pathway transcriptional activator and self-renewal agonist, leading to an R701G amino acid change, which stabilized GLI1 transcriptional activity by preventing the binding of a critical Hh signaling pathway negative regulator, and resulted in promotion of immunomodulatory drug resistance.^38^ Importantly, inactivation of ADAR1 reduced the resistance to blockade and overcame the resistance to immunotherapy.^39^

In the process of microRNAs (miRNAs) maturation, the stem-loop secondary structure adopted by primary transcripts of miRNA genes (pri-miRNAs) and miRNA precursors (pre-miRNAs) enable the interactions between the A-to-I editing machinery and the miRNA biogenesis pathway.^40^ ADARs can suppress miRNA maturation, which is another mechanism of gene regulation causing drug resistance.^41^ In leukemia, ADAR1-mediated miRNA editing impaired let-7 biogenesis and enhanced the self-renewal of leukemic stem cell (LSC). A small-molecule antagonizes ADAR1 on LSC self-renewal in stromal co-cultures restored let-7 biogenesis.^42^ Dihydrofolate reductase (DHFR) plays a key role in folate metabolism in cancers, and is a target of chemotherapeutic agents including methotrexate and pemetrexed. Upregulation of ADAR1 induced the expression of DHFR by editing the miR-25-3p, a miRNA targeting DHFR, which could enhance cellular proliferation and resistance to methotrexate.^43^ The 3’UTRs are important regions for posttranscriptional regulation mediated by miRNA. Modification of 3’ UTR sequence plays crucial role in cancer drug resistance. ADAR1-induced A-to-I editing stabilizes a proto-oncogene, mouse double minute 2 homolog (MDM2). Modulating the 3’ UTR region of MDM2 by ADAR1 can prevent the binding of miR-155, and enhance the reprogramming of progenitor cells into dormant leukemia stem cells.^44^ In some cases, A-to-I editing could also trigger abnormal splicing leading to drug resistance. A-to-I editing induced mis-splicing of GSK3β (Δ exon 9) in LSC, as a result, β-catenin expression was enhanced, leading to cancer progression and TKI resistance.^37,45^

#### mRNA methylation and cancer drug resistance

Over 100 different types of posttranscriptional RNA modifications have been documented among all living organisms so far.^46,47^ Although the majority of these modifications are found in tRNA and rRNA,^48^ increasing evidence have shown that the epigenetic modifications in messenger RNA (mRNA) are essential for almost every step of RNA biogenesis, physiology and turnover.^49^ Among them, N6 -methyladenosine (m^6^A) is the most prevalent modification that occurs in the mRNAs.^50^ The modification is catalyzed by the m^6^A methyltransferase “writers” and then recognized by m^6^A binding protein “readers”, and the m^6^A mark can be removed by demethylase “erasers”. The core m^6^A writer complex includes methyltransferase-like 3 (METTL3) and methyltransferase-like 14 (METTL14).^51^ The m^6^A erasers include fat mass and obesity-associated (FTO) and AlkB homolog 5 (ALKBH5) demethylases.^52,53^ The readers were firstly discovered to be YT521-B homology (YTH) domain-containing proteins (YTHDF1, 2, 3 and YTHDC1, 2).^51^ Soon afterward, more different types of readers were revealed.^54,55^ Interestingly, they exert diverse functions with different mechanisms.^55^ For example, YTHDF2 specifically recognizes and destabilizes m^6^A modified RNAs and re-localizes these RNAs to processing bodies; while YTHDF1 stimulates mRNA translation by interacting with translation initiation factors. The m^6^A marks are enriched at the RRACH (R = G or A, H = A, C, or U) motif around stop codons, 3’ or 5’ UTRs, and internal long exons.^51,56,57^ The m^6^A deposition is the beginning of the journey of methylation regulation. The m^6^A mRNA might be demethylated by erasers, or exported into cytoplasm and subject to be “read”. The dramatic and dynamic variations of mRNA modifications had been found in every step of biological processing, especially in tumor transformation and cellular reprogramming,^58^ implying their biological significance in cancer therapeutic treatment.

The biological significance of mRNA methylation is recently uncovered, however, the mechanism of regulation of m^6^A modification in drug resistance remains poorly understood. Most studies focused on the regulation of the transcripts that participated in the maintenance and modulation of the stemness and self-renewing CSCs that are thought to be responsible for complex tumor heterogeneity, cancer progression and therapeutic resistance.^59–61^ SOX2 is one of the major regulators in tumor initiation and cancer stem-cell functions.^62^ One Study showed that m^6^A writer METTL3 interacted with the 3′UTR of SOX2 mRNA and lead to methylation and stabilization of SOX2 mRNA in glioma stem-like cells (GSCs). The enhanced expression of METTL3 increased SOX2 expression, which maintained the stemness and radioresistance of GSCs.^63^ Interestingly, METTL3 is a multifunctional protein that not only has the activity of transmethylation, but can also regulate the mRNA translation in cytoplasm. In lung cancer, METTL3 promoted translation of oncogenic mRNA, epidermal growth factor receptor (EGFR), independent of its catalytic activity. METTL3 shuttled from nucleus to cytoplasm and interacted with ribosomes. Such interactions promoted EGFR mRNA translation leading to cell proliferation, survival, and invasion of cancers.^64^

The process of “reading” and “erasing” of m^6^A methylation marks are essential for regulation of genes that are responsible for drug resistance. In acute myeloid leukemia (AML), YTHDF2 is overexpressed and is required for disease initiation. Deletion of reader YTHDF2 compromises LSC development and propagation by increasing the half-life of tumor necrosis factor receptor 2 (TNFR2). Importantly, YTHDF2 is not essential for normal hematopoietic stem cells, indicating that YTHDF2 is a unique therapeutic target which specifically inhibits LSCs.^65^ In cervical cancer, FTO was found to induce DNA repair activity and drug resistance to chemoradiotherapy by increasing β-catenin mRNA through m^6^A methylation.^66^

Recently, inhibition of m^6^A eraser was found to be a potential strategy for overcoming drug resistance. Inhibition of m^6^A demethylases FTO and ALKBH5 was found to effectively overcome PARP inhibitor resistance in BRCA-mutated epithelial ovarian cancers.^67^ Mechanistically, deletion of m^6^A erasers increased FZD10 mRNA m^6^A modification and led to stabilization of FZD10 and upregulation of the Wnt/beta-catenin signaling pathway.^67^

In some cases, METTL3 promotes drug resistance through various pathways simultaneously. For example, METTL3 increased the mRNA of YAP, an effector of Hippo signaling pathway, leading to promotion of castration resistance in direct and indirect manners. On one hand, METTL3 prevented the degradation of YAP mRNA caused by miR-1914-3p via increasing the m^6^A modification of a noncoding RNA MALAT1 which was an RNA sponge of miR-1914-3p. On the other hand, METTL3 promoted YAP mRNA translation by recruiting YTHDF1/3 and EIF3b to the translation initiation complex machinery.^68^ Interestingly, a recent study revealed a crucial role of the m^6^A regulator in response to immunotherapy. Anti-PD-1 checkpoint blockade therapy had been proven as an important therapy for melanoma. However, more than 50% of patients didn’t show a durable response to immunotherapy. Inhibition of FTO increased m^6^A methylation in PD-1, leading to PD-1 mRNA decay through the m^6^A reader YTHDF2, eventually, sensitized melanoma to anti-PD-1 treatment. This study provides evidence suggesting that targeting m^6^A methylation regulator is a novel strategy for sensitizing immunotherapy.^69^

### Noncoding RNA modification in cancer drug resistance

**Fig. 2:**
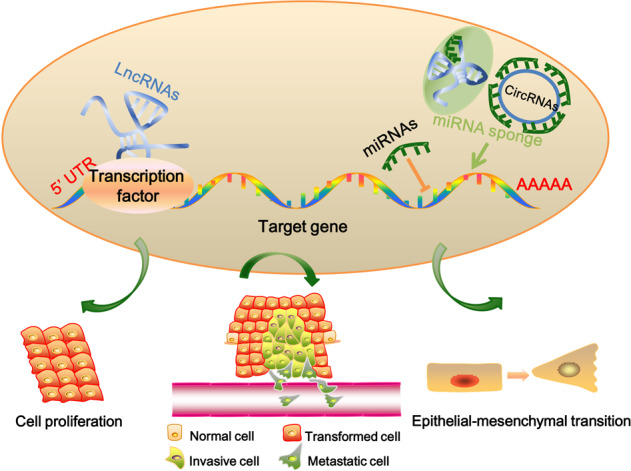
The functions of noncoding RNAs in cancer drug resistance. LncRNA can directly interact with target genes, or act as ceRNA to interact with miRNA to participate in gene expressions; circRNAs can act as “miRNA sponge” to release the inhibitory effect of miRNA on its target genes. The noncoding RNAs could be potential targets of drug resistance in cancers due to their functions in cell proliferation, metastasis, and EMT

The process of eukaryotic transcription refers to the genetic information being transformed from DNA to RNA.^70^ Subsequently, the message RNA (coding RNA) is translated into protein and the noncoding RNA performs function of posttranscriptional modification. The linear noncoding RNAs can be divided into three categories: miRNA, siRNA, and piRNA with the lengths of <50 nt; rRNA, tRNA, and snRNA with the lengths from 50 to 500 nt; and Long noncoding RNA (LncRNA) with the length of more than 500 nt.^71^ LncRNA can directly interact with target genes to activate or inhibit the expression of target genes. In addition, it can also act as competitive endogenous RNA (ceRNA) to interact with miRNA and participate in the regulation of gene expression.^72,73^ Because of the similar structures between lncRNA and mRNA, miRNA may negatively regulate lncRNA expression through a mechanism similar to mRNA.^74^ Unlike other linear RNAs, circular RNAs are closed circular structures, which lack the 5′ and 3′ ends and their expressions are more stable. Functional studies have revealed that circRNAs can release the inhibitory effect of miRNA on its target genes by acting as miRNA sponge.^75,76^ Currently, noncoding RNAs have attracted a lot of attention as potential targets of drug resistance in cancers due to their functions in cell proliferation, metastasis, and EMT (Fig. 2).^77–80^ In this section, we will focus on the role of miRNAs, LncRNAs, and circRNAs in chemoresistance.

#### miRNAs and cancer drug resistance

Recent studies have shown that miRNAs are involved in cisplatin (DDP)-mediated cancer drug resistance. Ubiquitin-conjugating enzyme E2C (UBE2C) is an active proto-oncogene and highly expressed in DDP-resistant NSCLC cells. miR-495 targeted 3′UTR of *UBE2C*, which acted as a transcriptional factor and downregulated the expression of cancer drug resistance associated genes such as *ABCG2* and *ERCC1*, thus reversing DDP resistance in NSCLC cells via reducing EMT, cell migration, and invasion.^81^ Another miRNA, miR-146b could bind to protein tyrosine phosphatase 1B, inhibit the EMT process, and reduce cisplatin resistance in human lung adenocarcinoma (LUAD) cells.^82^ Analysis of miRNA expression profiles and experiments in cisplatin-resistant and -sensitive ovarian cancer cells showed that miR-141 was overexpressed in cisplatin-resistant cells. miR-141 could directly target *KEAP1*, an oxidative stress regulator, and induced cisplatin resistance in ovarian cancer cells by activating NF-κB pathway.^83^ Exosomes are vesicles with a diameter of 40–100 nm. They play important roles in regulating tumor microenvironment, metastasis, and drug resistance by promoting transportation of mRNAs and miRNAs.^84,85^ The cancer-associated fibroblasts derived exosomal miR-196a accelerated head and neck cancer cell proliferation and cisplatin resistance through downregulating expression of target genes: *CDKN1B* and *ING5*.^86^ miR-936 suppressed cell proliferation, migration in glioma^87^, and non-small cell lung cancer,^88^ and induced drug resistance to cisplatin and DOX via targeting G protein-coupled receptor 78 (GRP78) in laryngeal squamous cell carcinoma cells.^89^

In addition, other EGFR-TKIs involved in drug resistance through regulating miRNAs expressions. Notch receptor 3 (NOTCH3) is highly expressed in LUAD and gefitinib-resistant cells. MiR-150 decreased IC_50_ of gefitinib, downregulated the expressions of target gene *NOTCH3*, which was positively correlated with collagen 1A1 expression, providing a potential therapeutic target for LUAD treatment.^90^ Bone morphogenetic protein 4 (BMP4) accelerates cancer cell energy metabolism and is upregulated in the EGFR-TKI-resistant cells. However, low-expression of miR-139-5p was found in TKI-resistant cells and combination of miR-139-5p and yuanhuadine significantly suppressed BMP4 expression and tumor growth in the resistant NSCLC cells and cell-derived xenograft (CDX) mouse model.^91^ Microarray analysis revealed that miR-214-3p was significantly decreased in multidrug resistant cells. MiR-214-3p directly targeted *ABCB1* and *XIAP*, promoted cell apoptosis, and sensitized retinoblastoma cells to multiple chemotherapeutic drugs. Overexpression of ABCB1 or XIAP could reverse chemoresistance induced by miR-214-3p.^92^ Tao et al. showed that miR-451a promoted the sensitivity of lung cancer cells to DOX via targeting c-myc to reduce expression of N-cadherin and Vimentin and enhance expression of E-cadherin.^93^ Han et al. revealed that miR-552 could promote the self-renewal, tumorigenesis. and sorafenib resistance via activating protein kinase B (AKT) phosphorylation in liver tumor-initiating cells (T-ICs).^94^

#### LncRNAs and cancer drug resistance

A number of studies have identified the function of lncRNAs in cancer drug resistance via various methods. A study using CRISPR activation of lncRNA system was developed to target 14,701 lncRNA genes in cytarabine-resistant acute myeloid leukemia and found that lncRNA GAS6-AS2 promoted cytarabine resistance via activating GAS6/TAM signaling pathway.^95^ Another study using whole-exome sequencing and transcriptional profiling in cetuximab-resistant cells in three-dimensional culture showed that lncRNA MIR100HG-derived miR-100 and miR-125b were overexpressed in cetuximab-resistant head and neck squamous cell carcinoma and colorectal cancer cells.^96^ RNA sequencing of drug-resistant and drug-sensitive NSCLC cells revealed that lncRNA ATP2B1 (or lncRNA HUWE1)-miR-222-5p-TAB could be the potential ceRNA regulatory network, which involved in drug resistance in NSCLC cells.^97^ LncRNA MSTRG51053.2 may act as the ceRNA for miR-432-5p in cisplatin resistance via targeting target genes such as *MGST1*, *MGST3*, *GST-ω1*, and *ABCG2* in NSCLC cells.^98^ LncRNA MALAT1-miR-22-3p-ZFP91 axis could promote oxaliplatin resistance in gastric cancer cells.^99^ LncRNA KCNQ1OT1 induced the chemoresistance to DOX in acute myeloid leukemia by targeting TSPAN3 through sponging miR-193a-3p.^100^

**Fig. 3:**
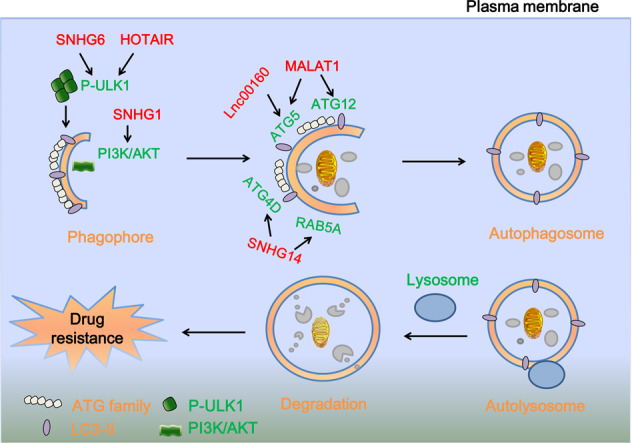
An illustration of the process of autophagy and the roles of lncRNAs in drug resistance via autophagy. The cells engulf and encapsulate cytoplasmic proteins or organelles into vesicles, and then vesicles fuse with lysosomes to form autophagy lysosomes, subsequently, autophagy lysosomes degrade the contents, recycle amino acids, fatty acids, and nucleotides. LncRNA MALAT1, and SNHG family could facilitate drug resistance via inducing autophagy and activating expressions of autophagy-related proteins

Autophagy is a process which can be divided into three phases: (1) cells engulf and encapsulate cytoplasmic proteins or organelles into vesicles; (2) vesicles fuse with lysosomes to form autophagy lysosomes; and (3) autophagy lysosomes degrade the contents, recycle amino acids, fatty acids, and nucleotides.^101,102^ Eventually, autophagy achieves the goal of renewal of damaged cell organelles, misfolded proteins, and provides nutrients and energy for cells.^103,104^ Some studies have shown that lncRNAs accelerate cancer drug resistance via modulating the expressions of autophagy-associated genes. Knockdown of LncRNA-HOTAIR could promote the sensitivity of crizotinib in NSCLC cells via suppressing autophagy and ULK1 phosphorylation.^105^ LncRNA LINC00160 upregulated the expressions of autophagy-associated proteins such as LC3I/II and ATG5, and then induced autophagy and drug resistance in hepatocellular carcinoma cells via miR-132-PIK3R3 axis.^106^ LncRNA MALAT1 induced autophagy and facilitated the resistance to cisplatin in gastric cancer via miR-30b-ATG5^107^ or miR-23b-3p-ATG12^108^ or in hepatocellular carcinoma cells via HIF-2α-MALAT1-miR-216b.^109^ The lncRNA SNHG family has recently been implicated in the modulation of drug resistance. SNHG6-miR-26a-5p-ULK1 axis could promote colorectal cancer cell resistance to 5-fluorouracil through inducing autophagy.^110^ Another member of SNHG family, lncRNA SNHG1 also contributed to sorafenib resistance by activating AKT pathway and inducing autophagy via sponging miR-21 in hepatocellular carcinoma cells.^111^ In addition, lncRNA SNHG14 induced the expressions of autophagy-related proteins such as RAB5A and ATG4D via sponging miR-101 and then enhanced the gemcitabine resistance in pancreatic cancer (Fig. 3).^112^

#### CircRNAs and cancer drug resistance

Circular RNA can relay the inhibitory effect of miRNA on its target genes by acting as miRNA sponge. There is a growing evidence that circRNAs accelerate cancer drug resistance.^113^ CircAKT3 is highly expressed in cisplatin-resistant gastric cancer cells. Huang et al. showed that circAKT3 upregulated PIK3R1 expression via sponging miR-198 and promoted cisplatin resistance in gastric cancer.^114^ CircPAN3 induced DOX resistance in acute myeloid leukemia via regulating autophagy-associated AMPK/mTOR signaling pathway and protein expressions of LC3I/II and p62^115^ or miR-153-5p/miR-183-5p-XIAP axis.^116^ Hsa_circ_0079662 interacted with hsa-miR-324-5p as the ceRNA and enhanced the resistance to oxaliplatin via TNF-α pathway in human colon cancer.^117^ Hsa_circ_0060060 accelerated expressions of autophagy marker LC3 and p62 through miR-144-3p/TGF-α axis and promoted cisplatin resistance in papillary thyroid carcinoma and anaplastic thyroid carcinoma cells.^118^ In addition, circCELSR1 was upregulated in paclitaxel-resistant ovarian cancer cells. Inhibition of circCELSR1 enhanced paclitaxel-induced cytotoxicity via upregulating FOXR2 expression or acting as the sponge for miR-1252 and increased cell apoptosis.^119^ Dong et al found that circ_0076305 was upregulated in NSCLC and promoted cisplatin resistance in NSCLC by upregulating STAT3 via targeting miR-296-5p.^120^

However, some studies indicated that circRNAs increased cancer drug sensitivity. For example, Li et al. showed that circ_0002483 enhanced paclitaxel sensitivity in NSCLC by targeting *GRB2*, *FOXO1*, and *FOXO3* via miR-182-5p.^121^ Sang et al. found that Hsa_circ_0025202 could inhibit tumor progression and enhance the sensitivity of cancer cells to tamoxifen in breast cancer via targeting miR-182-5p/FOXO3a axis.^122^ Moreover, Liang et al. indicated that decreased expression of circKDM4C in breast cancer suppressed DOX resistance through miR-548p/PBLD pathway.^123^ The roles and the molecular mechanisms of noncoding RNAs in cancer drug resistance are outlined in Table 2.

**Table 2:** Noncoding RNAs in cancer drug resistance

Noncoding RNA	Target gene	Cancer	Function	Drug	Reference
miR-495	ABCG2, ERCC1	Lung cancer cells	Drug sensitivity	Cisplatin	^81^
miR-146b	PTP1B	Lung cancer cells	Drug sensitivity	Cisplatin	^82^
miR-141	KEAP1	Ovarian cancer	Drug resistance	Cisplatin	^83^
miR-196a	CDKN1B, ING5	Head and neck cancer	Drug resistance	Cisplatin	^86^
miR-936	GPR78	Laryngeal squamous cell carcinoma	Drug resistance	Cisplatin	^89^
miR-150	NOTCH3	Lung adenocarcinoma	Drug sensitivity	Gefitinib	^90^
miR-214-3p	ABCB1, XIAP	Retinoblastoma cells	Drug sensitivity	Multiple chemodrugs	^92^
miR-451a	N-cadherin, Vimentin and E-cadherin	Lung cancer cells	Drug sensitivity	Doxorubicin	^93^
miR-552	PTEN	Liver tumor-initiating cells	Drug resistance	Sorafenib	^94^
LncRNA MIR100HG	GATA6	Colorectal cancer and head and neck squamous cell cancer	Drug resistance	Cetuximab	^96^
LncRNA MSTRG51053.2	MGST1, MGST3, GST-ω1, ABCG2	Lung cancer	Drug resistance	Cisplatin	^98^
LncRNA MALAT1	ZFP91, ATG5, ATG12, HIF-2α	Gastric cancer and hepatocellular carcinoma cancer	Drug resistance	Oxaliplatin, Cisplatin	^99,107–109^
LncRNA KCNQ1OT1	TSPAN3	Acute myeloid leukemia	Drug resistance	Adriamycin	^100^
LncRNA-HOTAIR	ULK1	Lung cancer	Drug resistance	Crizotinib	^105^
LncRNA LINC00160	PIK3R3	Hepatocellular carcinoma cancer	Drug resistance	Sorafenib	^106^
LncRNA SNHG6	ULK1	Colorectal cancer	Drug resistance	5-fluorouracil	^110^
LncRNA SNHG14	RAB5A and ATG4D	Pancreatic cancer	Drug resistance	Gemcitabine	^112^
CircAKT3	PIK3R1	Gastric cancer	Drug resistance	Cisplatin	^114^
CircPAN3	LC3I/II, p62 and XIAP	Acute myeloid leukemia	Drug resistance	Doxorubicin	^115,116^
Has_circ_0079662	HOXA9	Colon cancer	Drug resistance	Oxaliplatin	^117^
Hsa_circ_0060060	TGF-α	Papillary thyroid carcinoma and Anaplastic thyroid carcinoma cancer	Drug resistance	Cisplatin	^118^
CircCELSR1	FOXR2	Ovarian cancer	Drug resistance	Paclitaxel	^119^
Circ_0076305	STAT3	Lung cancer	Drug resistance	Cisplatin	^120^
Circ_0002483	GRB2, FOXO1, and FOXO3	Lung cancer	Drug sensitivity	Taxol	^121^
Hsa_circ_0025202	FOXO3a	Breast cancer	Drug sensitivity	Tamoxifen	^122^
CircKDM4C	PBLD	Breast cancer	Drug sensitivity	Doxorubicin	^123^

*ABCG2* ATP-binding cassette subfamily G member 2, *ERCC1* ERCC excision repair 1, endonuclease non-catalytic subunit, *PTP1B* protein tyrosine phosphatase non-receptor type 1, *KEAP1* kelch like ECH associated protein 1, *CDKN1B* cyclin dependent kinase inhibitor 1B, *ING5* inhibitor of growth family member 5, *GPR78* G protein-coupled receptor 78, *NOTCH3* notch receptor 3, *ABCB1* ATP-binding cassette subfamily B member 1, *XIAP* X-linked inhibitor of apoptosis, *PTEN* phosphatase and tensin homolog, *GATA6* GATA binding protein 6, *MGST1* microsomal glutathione S-transferase 1, *MGST3* microsomal glutathione S-transferase 3, *ZFP91* ZFP91 zinc finger protein, *ATG5* autophagy-related 5, *ATG12* autophagy related 12, *TSPAN3* tetraspanin 3, *ULK1* unc-51 like autophagy activating kinase 1, *PIK3R3* phosphoinositide-3-kinase regulatory subunit 3, *ATG4D* autophagy related 4D cysteine peptidase, *PIK3R1* phosphoinositide-3-kinase regulatory subunit 1, *XIAP* X-linked inhibitor of apoptosis, *HOXA9* homeobox A9, *TGF-α* transforming growth factor alpha, *FOXR2* forkhead box R2, *STAT3* signal transducer and activator of transcription 3, *GRB2* growth factor receptor bound protein 2, *FOXO1* forkhead box O1, *FOXO3* forkhead box O3, *PBLD* phenazine biosynthesis like protein domain containing

### Protein modification in cancer drug resistance

**Fig. 4:**
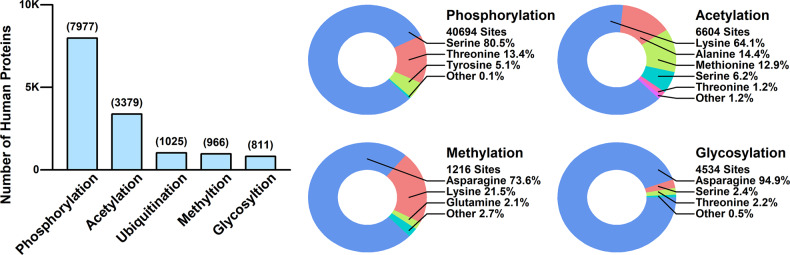
The PTM status of human proteins. All data is retrieved from Uniprot database and updated as of 2015-05. The number of proteins with different types of PTMs are illustrated in the barplot (left); the percentages of amino acids modified in each type of PTMs are illustrated in the circle plots (right)

PTM refers to the enzymatic modification after the biosynthesis of proteins, which is crucial for regulating and maintaining the functions of proteins. A large portion of human proteins have gone through at least one round of PTM after being synthesized. The PTM status of human proteins retrieved from Uniprot database (www.uniprot.org) is summarized and illustrated in Fig. 4. Among different types of PTMs, phosphorylation is the most common one, with 7977 human proteins containing 40,694 phosphorylation sites, and serine is the most common phosphorylated amino acid. Among these 7977 proteins, the top-five-enriched GO biological processes are “organelle organization,” “cell localization,” “regulation of cellular component organization,” “positive regulation of metabolic process,” and “establishment of localization in cell.” Acetylation ranks the second, with 3379 human proteins containing 6604 acetylation sites, and lysine is the most preferred acetylation amino acids. Among these 3379 proteins, the top-five-enriched GO biological processes are “organelle organization,” “cellular catabolic process,” “mRNA metabolic process,” “catabolic process,” and “organic substance catabolic process.” Ubiquitination ranks the third, with 1025 proteins being ubiquitinated. Among these 1025 proteins, the top-five-enriched GO biological processes are “cellular protein modification process,” “protein modification process,” “macromolecule modification,” “protein modification by small protein conjugation or removal,” “positive regulation of metabolic process.” Methylation ranks the fourth, with 966 proteins containing 1216 methylation sites, and asparagine is the most ordinary methylation amino acid. Among these 966 proteins, the top-five-enriched GO biological processes are “mRNA metabolic process,” “organelle organization,” “small GTPase-mediated signal transduction,” “mRNA processing,” and “Ras protein signal transduction.” Glycosylation ranks the fifth, with 811 proteins containing 4534 glycosylation sites, and asparagine is the most preferred glycosylation amino acid. Among these 811 proteins, the top-five-enriched GO biological processes are “biological adhesion,” “cell adhesion,” “extracellular structure organization,” “immune system process,” and extracellular matrix organization.” The mechanisms of PTMs in chemoresistance can either be direct, in which the modifications disrupt binding sites; or indirect, in which upstream modifications lead to pathway blockage. In this section, we will briefly discuss the role of PTM in chemoresistance from the perspectives of drug inactivation/efflux, drug target modifications, DDR, cell death resistance, EMT and metastasis. Some of the reported protein targets causing chemoresistance are listed in Table 3.

**Table 3:** List of protein targets in chemoresistance

Proteins	Drugs	Reference
CES, CDA, DPD	5-FU	^128,129,131^
P-glycoprotein	Anthracycline, daunorubicin, epipodophyllotoxins	^134–136^
EGFR	Gefitinib, erlotinib, cetuximab	^146,147^
HER2	Herceptin	^150,151^
HER3	Gefitinib	^154^
HER4	Lapatinib	^155^
p65, Pin1	Doxorubicin	^170^
BCL, Caspase 3	Doxorubicin	^174^
CRL4, BIRC3	Cisplatin	^175^
E-cadherin	Erlotinib	^183^

*CES* carboxylesterases, *CDA* cytidine deaminase, *DPD* dihydropyrimidine dehydrogenase, *EGFR* epidermal growth factor receptor, *HER2* erb-b2 receptor-tyrosine kinase 2, *Pin1* peptidylprolyl cis/trans isomerase, NIMA-interacting 1, *CRL4* culling-ring ubiquitin ligase 4, *BIRC3* baculoviral IAP repeat containing 3

#### Drug inactivation

**Fig. 5:**
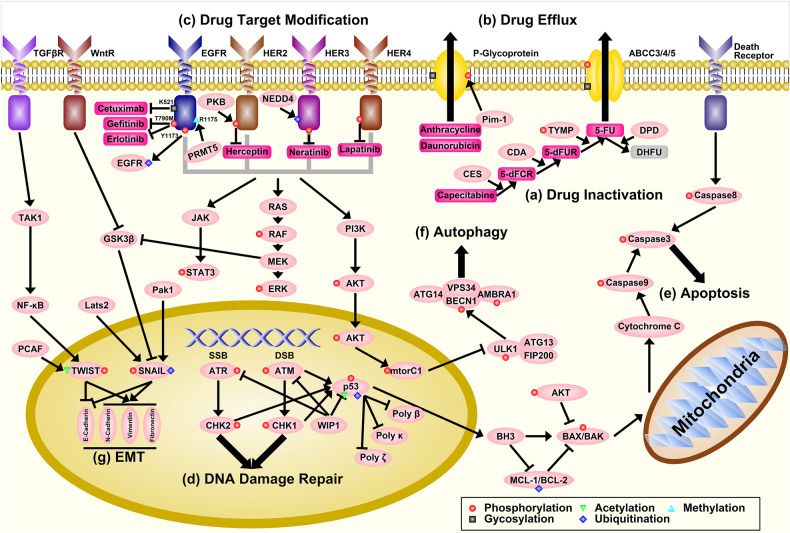
The mechanisms of PTMs in cancer cell chemoresistance. **a** The inactivation of Capecitabine through the regulation of CES, CDA, TYMP, and DPD enzymes. **b** The drug efflux process mediated by ABC transporter proteins and the modifications of these transporters. **c** The modification of ERBB receptors through mutations and PTMs resulting in multiple drug resistance. **d** DNA damage repair system in cancer cells could also result in drug resistance, and this process is mediated through the repression of ATM and ATR, as well as p53 proteins, and induction of specialized DNA polymerases, such as Poly beta, kappa and zeta. **e** The repression of apoptosis in cancer cells, which is mainly achieved through the inhibition of p53 via either mutation or PTMs. The overexpression of MCL-1/BCL-2 and repression of BAX/BAK proteins also contribute to this process. **f** The repression of autophagy in cancer cells. MTORC1 is triggered through PI3K-AKT pathways, which further inhibits the phosphorylation of ULK1, and impedes the autophagy process. **g** The activation of EMT in cancer cells. EMT process is triggered through multiple signaling pathways including TGFβR and WntR, which activate SNAIL and TWIST transcription factors. These EMT-TFs repress the expression of E-cadherin and promote the expression of N-cadherin, vimentin, and fibronectin, which further promote EMT process

Certain drugs need metabolic modification to become their active forms, and some cancer cells have developed the ability to modify/shut down these activation processes or to use other processes to deactivate the active forms of drugs. For example, capecitabine, under the brand name Xeloda, is a widely used chemotherapeutic drug in the treatment of breast cancer, gastric cancer, and colorectal cancer.^124^ Inside the human body, capecitabine needs to be metabolized into 5-FU, a thymidylate synthase inhibitor which interrupts the process of pyrimidine thymidine synthesis.^125^ This process is mediated through three enzymes, carboxylesterases (CES), cytidine deaminase (CDA), and thymidine phosphatase (TYMP), as detailed in Fig. 5a. TYMP is a dual-role enzyme in cancer development and treatment: on one side, after phosphorylated by protein kinase C, TYMP is capable of converting doxifluridine (5-dFUR), an intermediate product of capecitabine, to its active form, 5-FU, which is effective in cancer treatments;^126^ on the other side, TYMP has been found as a pro-oncogene in many studies, where it is overexpressed in many types of cancers, and the overexpression of TYMP promotes tumor angiogenesis and inhibits apoptosis.^127^ As for CES and CDA, although there havn’t been any direct evidence, the changes of catalytic activity of CES and CDA genes in some cancer patients with SNPs might be related to PTMs.^128,129^ Aside from mutations, CES is also positively regulated by p53, a well-known tumor repressor, which is often mutated or posttranslational modified in cancer cells (will be discussed in the following context),^130^ hence the downregulation of CES expression in cancers can result in chemoresistance.

Aside from impeding the activation process, deactivation also contributes to drug resistance. For example, dihydropyrimidine dehydrogenase (DPD) is a key enzyme in pyrimidine catabolism,^131^ and is capable of reducing the pyrimidine double bonds of 5-FU, and converting it to dihydrofluorouracil. This process deactivates 5-FU and also results in chemoresistance. In certain type of cancer (e.g., head and neck squamous cell carcinoma), DPD is overexpressed, and overexpression of DPD is one of the main reasons causing 5-FU resistance.^132^

#### Drug efflux

Efflux is the process of moving a variety of compounds out of cells, which is mediated by some ATP-binding cassette (ABC) transporters, which are also named multiple drug resistance (MDR) proteins. Among them, P-glycoprotein, encoded by ABCB1 (MDR1), is one of the most widely studied transporters.^133^ P-glycoprotein is highly expressed in many drug-resistant tumors and is involved in the efflux of many anticancer drugs such as anthracycline, daunorubicin, epipodophyllotoxins, and others (Fig. 5b).^134–136^ The regulation of P-glycoprotein is explored in many studies: Takada et al. showed that the expression of P-glycoprotein was positively regulated by MAPFK signaling pathways in human breast cancer;^137^ Henrique et al. found that ABCB1 was epigenetically regulated through posttranslational histone modification in prostate cancer;^138^ Xie et al. revealed that Pim-1 could phosphorylate P-glycoprotein, which protects P-glycoprotein from degradation and enables its glycosylation.^139^ Overexpression of Pim-1 in many human cancers indirectly contributes to P-glycoprotein-mediated drug-efflux and chemoresistance.^140^ The Pim-1 inhibitor SGI-1776 was reported to overcome P-gp-mediated drug resistance.^141^

In terms of 5-FU, although there have been activation and deactivation processes in many cancer types, drug efflux also contributes to the chemoresistance (Fig. 5b). 5-FU and its downstream product fluorodeoxyuridylate (FdUMP) can be pumped out of cells through several transporters such as ABCC3/4/5/11.^142–144^ PTMs play important roles in these ABC transporter-mediated drug resistance, e.g., phosphorylation directly affects the efficiency of transporters;^145^ glycosylation influences the stability of transporters and protects them from proteases.^146^

#### Drug target modification

Most of the cancer drugs aim for some specific proteins, causing structural changes of targets, leading to the blockage of certain pathways, and resulting in death of cancer cells. To resist these effects, many cancer cells alter the target proteins either by decreasing/halting their expression or modifying their structures to hinder the binding process (Fig. 5c). For example, EGFR, one of the most intensively studied receptor-tyrosine kinases (RTKs), was found to have a variety of mutations and PTMs that resulted in multiple chemoresistance in many different types of cancers: T790M mutation was directly related to gefitinib and erlotinib resistance in non-small cell lung cancer;^147^ lack of glycan sialyation due to K521 polymorphism resulted in cetuximab resistance in head and neck cancers;^148^ methylation of R1175 by PRMT5 modulated EGF-induced phosphorylation at W1173, which further promotes ERK activation in breast cancer;^149^ SRC kinase-mediated EGFR ubiquitination, and degradation in colorectal cancer.^150^ Aside from EGFR, other ErbB family members also showed alterations in many cancers. Phosphorylation by PKB inhibited the activation of HER2 in breast cancer and resulted in resistance to herceptin;^151,152^ neural precursor cell expressed and developmentally downregulated 4, an E3 ubiquitin ligase, mediated the degradation of HER3 in prostate cancer through ubiquitination.^153^

Cancer cells could also use alternative pathways to bypass or compensate for drug actions. For example, VanMeter et al. revealed an alternative mechanism of downstream protein activation, which is independent of EGFR in non-small cell lung cancer;^154^ Sergina et al. showed that overexpression of HER3 could lead to a compensatory shift in phosphorylation-dephosphorylation equilibrium, resulting in gefitinib resistance;^155^ Canfield et al. found that HER4 was upregulated in HER2+ breast cancer with lapatinib resistance, and knockdown of HER4 significantly decreased ATK phosphorylation levels, indicating the bypassing receptor function of HER4 in lapatinib-resistant cells.^156^

#### DNA damage repair (DDR)

Many cancer drugs can cause direct or indirect DNA damages, and the removal of these DNA lesions leads to cell survival. DDR system could induce cell-cycle arrest, leading to DNA repair, which protects the cell or leads to cell apoptosis and results in cell death (Fig. 5d). Increasing DNA repair and damage tolerance as well as evading apoptosis were the potential mechanisms of chemotherapy resistance in cancer cells.^157^ After chemotherapeutic treatment, DDR could induce a rapid but faulty repair mechanism, and the tolerance of DNA damages were achieved through several specialized DNA polymerases, such as poly beta, kappa, and zeta, that had low fidelity in DNA duplication and resulted in mutations.^158,159^ The overexpression of these specialized polymerases in chemoresistant cancers has been revealed in many studies. For example, elevated expression of Poly beta had been found in drug-resistant cancer cells,^160^ and knockdown of Poly beta by siRNA resensitized cancer cells to cisplatin;^159^ upregulated poly kappa had also been examined in lung cancer^161^ and inactivation of p53 promoted the expression of poly kappa.^162^ The genomic instability caused by tolerance of mutations was one of the main features of cancer.^163^

Ataxia-telangiectasia-mutated (ATM) and ataxia-telangiectasia and Rad3-related (ATR) kinases are activated by stresses of DNA double strand breaks and single strand breaks. Activation of ATM/ATR induces cell-cycle arrest (through CHK1 and CHK2) and cell apoptosis (through p53). In many cancer cells, the expression of ATM/ATR is downregulated^164^ and the activity of ATM/ATR is also reduced by upregulating WIP1 phosphatase, which dephosphorylates ATM/ATR and its substrates, e.g., p53.^165^ Interestingly, in certain types of cancers, where ATM/ATR is uncoupled from cell-cycle arrest and apoptosis, the overexpression of ATM/ATR has been found in many studies, which proves the importance of ATM/ATR in chemoresistance.^166^

#### Cell death resistance

A significant hallmark of cancer cells is the ability of resisting cell death,^167^ hence evading apoptosis and autophagy is one of the most important abilities of cancer cells. In normal cells, apoptosis is induced either through extrinsic or intrinsic signaling pathways. In cancer cells, the components in both extrinsic and intrinsic signaling pathways are either mutated or mis-regulated, therefore apoptosis process is impeded (Fig. 5e). For example, extrinsic apoptosis pathways were triggered by surface death receptors such as FAS, DR4/5, and in many cancers those death receptors were often mutated or PTM modified,^168^ that greatly impeded apoptosis process. Moreover, some decoy receptors such as TRID and TRUNDD were overexpressed in certain cancers,^169^ which repressed extrinsic signaling pathway-induced apoptosis. Intrinsic signaling pathways are mainly induced by p53, which is often mutated or modified in cancer cells, as reviewed in Mansoori et al.,^170^ resulted in inhibiting the intrinsic apoptosis process from very beginning. Aside from p53 mutation, p65 subunit of nuclear factor-kappa B, is one of the regulators of tumorigenesis, and the suppression of p65 signaling could enhance the DOX-induced apoptosis in cervical cancer.^171^ MCL-1/BCL-2, the anti-apoptotic proteins, was found to be overexpressed in many types of cancers.^172,173^ BAX, the pro-apoptotic protein, could be phosphorylated by AKT at residue 184 in breast cancer, which prevented it from entering into mitochondria, resulting in chemoresistance.^174^ Caspase 3, the executioner of apoptosis, was phosphorylated by p38-MAPK, which was negatively regulated by TYMP, and the overexpression of TYMP in many cancers helped cancer cell to evade apoptosis and contributed to chemoresistance.^127^ Jaime-Sánchez et al.^175^ showed that EL4 cells overexpressing Bcl-X_L_ or DNC3 (a dominant negative form of caspase 3) proteins exhibited multidrug resistance such as DOX, and these EL4 cells could be eliminated by antigen-specific primed cytotoxic T cells. Recently, Hu et al.^176^ have showed that culling-ring ubiquitin ligase 4 (CRL4) could regulate the expression of BIRC3 (one of the inhibitors of apoptosis proteins) through STAT3 pathway, and BIRC3 is associated with cisplatin-resistance in ovarian cancer cells, suggesting the potential functional role of CRL4 and BIRC3 as novel therapeutic targets for cisplatin-resistant patients.

Autophagy suppresses tumor cells via lysosomal degradation pathway, and this process is mediated through ULK1 complex and VPS34 complex. ULK1 functions in complex with ATG13 and FIP2000,^177^ which further phosphorylates AMBRA1 and BECN1 and leads the translocation of VPS34 complex to ER and initiates autophagy process.^178^ In cancer cells, MTORC1 is activated through the PI3K-AKT pathway. Activated MTORC1 phosphorylates ULK1 on S758, which inhibits the function of ULK1 and hence impedes autophagy process. Moreover, mutations in MTOR, as well as upstream or downstream signaling components, conferring constitutive activation of MTOR signaling have also been reported in many studies.^179,180^

#### EMT and cancer metastasis

EMT refers to the process of converting epithelial cells into mesenchymal cells. Tumor metastasis is the process of primary tumor cells entering the bloodstream or lymph vessels and settling at other sites. Classically, EMT is considered as a promoter of metastasis, during which cancer cells acquire mobility and the capacity to migrate away from the primary site.^181^ EMT is triggered and regulated by a complex network as summarized in Thiery and Sleeman^182^ and executed by EMT transcription factors (EMT-TFs) consisted of SNAIL and TWIST family members.^183^ These EMT-TFs repress the expression of E-cadherin, which is a glycoprotein helping in epithelial cell anchorage, and stimulate cells to gain mesenchymal markers, such as N-cadherin, vimentin, and fibronectin.

One of the main mechanisms of EMT-mediated chemoresistance is through E-cadherin. Thomson et al.^184^ found that NSCLC cells with E-cadherin expression showed greater sensitivity to EGFR kinase inhibition (such as erlotinib), and cells overexpressed vimentin/fibronectin after EMT were insensitive to EGFR inhibition. Many EMT-TFs, such as SNAIL1, SNAIL2, and TWIST, possess the ability of repressing the expression of E-cadherin. The activity of these TFs is mainly regulated through a number of PTMs. For example, Snail was phosphorylated by GSK3β, which led to its cytosolic localization and β-Trcp-mediated ubiquitination.^185^ SNAIL was phosphorylated by LATS2 and PAK1, which promoted its nuclear transport and increased its stability when GSK3β was inhibited by RTK or WntR signaling pathways.^186,187^ Similarly, TWIST was acetylated by p300/CBP-associated factor, which regulated its intracellular location and transcriptional activity in urothelial cancer cells.^187^

Besides E-cadherin, EMT-TFs could induce chemoresistance through other intermediates. For example, TWIST could transcriptionally upregulate the expression of AKT2, which induced paclitaxel resistance in breast cancer, and this resistance was reduced with the silence of AKT2;^188^ TWIST could also repress the expression of estrogen receptor-α (ER) together with histone deacetylase 1 in breast tumors, which might contribute to the hormone-resistance in ER-negative breast cancer.^189^

## Epitranscriptomic and epiproteomic modifications as therapeutic targets

### Epitranscriptomic modifications

**Fig. 6:**
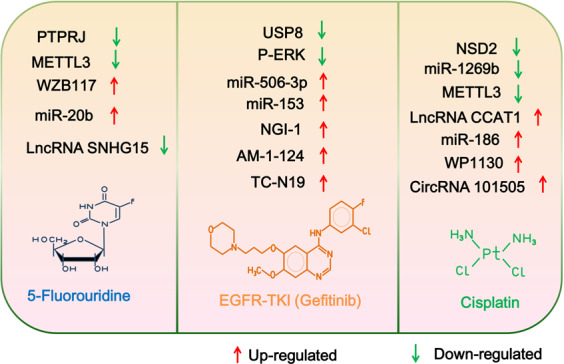
Epitranscriptomic and epiproteomic modifications could be the potential therapeutic targets in cancers. The critical genes and proteins in both modifications could reverse the resistance of cancer cells to chemotherapeutic drugs such as 5-fluorouridine, EGFR-TKI, and cisplatin

The roles of mRNA and noncoding RNA modification in drug resistance indicated that epitranscriptomic modifications could be the potential therapeutic targets in cancers (Fig. 6). Knockdown of METTL3 in pancreatic cancer showed significantly increased sensitivity to drugs such as 5-fluorouracil, cisplatin, gemcitabine, and irradiation.^190^ The downregulation of m^6^A demethylases FTO and ALKBH5 increased FZD10 m^6^A methylation and reduce sensitivity to PARPi in BRCA-mutated epithelial ovarian cancers.^67^ Zhu et al. showed that expression of miR-506-3p was decreased in gefitinib-resistant PC-9R cells, and miR-506-3p mimic could reverse the resistance of NSCLC cells to gefitinib through targeting YAP1.^191^ MiR-1269b directly targeted PTEN to activate PI3K/AKT signaling pathway and miR-1269b inhibitor could overcome cisplatin resistance in human NSCLC cells, speculating that miR-1269b could be a potential target for treatment of NSCLC.^192^ MiR-186 decreased TWIST1 expression via reversing mesenchymal-to-epithelial transition phenotype and resensitized ovarian cancer cells to cisplatin.^193^ Another miRNA, miR-20b could target ADAM9 to decrease the 5-FU resistance in colon cancer.^194^ MiR-153 directly targeted ABCE1 to inhibit gefitinib resistance in lung cancer, which may provide a therapeutic target to reverse the resistance to gefitinib in the future.^195^

LncRNA CCAT1 was overexpressed in esophageal cancer and its knockdown significantly repressed the tumor growth and promoted the sensitivity of cisplatin via miR-143/PLK1/BUBR1 axis, which suggested that CCAT1 could be a potential therapeutic target to overcome the cisplatin resistance in esophageal cancer.^196^ The depletion of lncRNA UCA1 attenuated the activity of Wnt/β-catenin signaling pathway and increased the tamoxifen sensitivity in breast cancer.^197^ Knockdown of lncRNA XIST could sensitize colorectal cancer to DOX via miR-124/SGK1 axis, which indicated that lncRNA XIST could be a potential therapeutic target to overcome chemoresistance in colorectal cancer.^198^ Silencing to lncRNA SNHG15, a bifunctional MYC-regulated noncoding RNA, could inhibit the cancer progression in CRC cells and promote the sensitivity of CRC cells to 5-FU, indicating that lncRNA SNHG15 could be a potential prognostic marker for chemotherapy.^199^ Expression of circRNA_101505 was downregulated in cisplatin-resistant hepatocellular carcinoma cells, and cisplatin toxicity was enhanced by circRNA_101505 sponging miR-103/NOR1 pathway.^200^

### Epiproteomic modifications

Various studies have indicated that targeting epiproteomic modifications can ameliorate drug resistance in cancers (Fig. 6). Histone methyltransferase SMYD2 promoted tumor progression in renal cell carcinoma, and combination treatment with SMYD2 inhibition and anticancer drugs significantly reduced the tumor volumes and weights, suggesting that SMYD2 could be a potential target for RCC treatment.^201^ PI3K/mTOR inhibitor and EGFR repression played the coordinated role in animal survival and gefitinib-targeted therapy in malignant glioma.^202^ NGI-1, an inhibitor of oligosaccharyltransferase could block the interaction between MET and EGFR, resulting in increasing sensitivity to gefitinib and osimertinib in EGFR mutated NSCLC cells.^203^ AM-1-124 specifically targeted STAT3 and downregulated STAT3 phosphorylation overcame drug resistance in TKI-resistant chronic myeloid leukemia cells.^204^ TC-N19, which is the dual inhibitor of EGFR and cMET, degraded both proteins via ubiquitin proteasome pathway and overcame gefitinib resistance in NSCLC cells.^205^ Qi et al. found that the phosphorylation of ERK increased in gefitinib-resistant NSCLC cells and the inhibition of ERK phosphorylation reversed gefitinib resistance via suppressing autophagy in lung cancer.^206^ Knockdown of ubiquitin-specific peptidase 8 (USP8) decreased the phosphorylation of EGFR, c-MET, ERBB2, and ERBB3, and a synthetic USP8 inhibitor displayed a smaller tumor size and a reversed gefitinib resistance in H1975 CDX model.^207^ WZB117, a specific inhibitor of Glut1, significantly increased the 5-FU resistance and could be used as the potential treatment in patients with 5-FU-resistant colon cancers.^208^ Protein tyrosine phosphatase receptor J (PTPRJ) was downregulated in human cervical tumor tissues and inhibition of PTPRJ could have promoted the resistance to 5-FU through activating JAK1/STAT3 pathway.^209^ Histone methyltransferase NSD2 mediated BCL-2 and SOX2 H3K36me2 modification and activated the levels of p-ERK and p-AKT in osteosarcoma. Knockdown of NSD2 induced cell apoptosis and led to the enhancing sensitivity of osteosarcoma to cisplatin.^210^ WP1130, the USP9x inhibitor, induced the degradation of transcription factor PBX1 and accelerated cell apoptosis in prostate cancer, which provided a new idea for prostate cancer treatment.^211^

## Conclusions and future perspectives

Epigenetic alternations in epitranscriptomic and epiproteomic modifications play critical roles in cancer treatment and drug resistance. Future researches on the function of epigenetics in vivo and its effect on conquering drug resistance are warranted. For example, even though emerging evidences have indicated that m^6^A regulators play important roles in cancer drug resistance by modulating the epitranscriptome, the research on m^6^A methyltransferase family is mainly focused on METTL3 and METTL14, and the underlying molecular mechanisms of other m^6^A methyltransferase members in drug resistance need to be further investigation. Recently, miRNAs have been discovered in exosomes, a structure that contained abundant genetic information and widely distributed in various body fluids. The potential advantages of miRNAs in exosomes imply that the roles in cancer drug resistance are worthy explored. The mutations related to cancer drug resistance have been identified, such as EGFR T790M and C797S. However, the relationship between mutations and protein expressions is not fully consistent. Temporal proteomic is being used as an emerging technology to target drug resistance and researchers showed that the combination of KRASi and HSP90 inhibitor (17-AAG) or cell-cycle inhibitor (CDK4/6i) could block cell growth and inhibit cancer drug resistance,^212,213^ which suggested that proteomic can be used as an effective treatment strategy for overcoming cancer drug resistance. Overall, targeting epigenetic alternations may improve cancer treatment and provide new approaches in overcoming drug resistance.

The dynamic intratumoral heterogeneity and the increased clonal repopulation are the main causes of cancer acquired resistance to platinum-based chemotherapy. Single-cell RNA-seq (scRNA-seq), the technology which allows transcriptomic analysis in individual cells, can dissect the heterogeneity and subpopulations in tumor microenvironment during cancer drug resistance.^214^ More attempts have been made to reveal the mechanism of drug resistance in cancers by scRNA-seq.^215–217^ For example, scRNA-seq from paclitaxel-sensitive and -resistant esophageal squamous cell carcinoma (ESCC) identified the subpopulations of paclitaxel-resistant ESCC cells, and research on the mechanism revealed the carfilzomib, a proteasome inhibitor, could reverse the paclitaxel resistance via activating the HIF-1 pathway.^218^ The transcriptome mapping of cisplatin-resistant tumor cells by scRNA-seq uncovered a novel gene COX7B, and inhibition of COX7B reduced the sensitivity of cisplatin, which provided the valuable insights into chemosensitivity in cancers.^219^

An era of single-cell omics has arrived, and the future clinical applications based on epitranscriptomics and epiproteomics are very promising: (i) single-cell multiple omics sequencing technique can be used widely to analyze the transcriptome, proteome, epitranscriptome, and epiproteome simultaneously at the single-cell level in drug resistance cancer cells, which allows us to reveal the unknown mechanisms and targets;^220,221^ (ii) personalized single-cell sequencing provides comprehensive clues to optimize the therapeutic strategy against relapsing cancers;^222^ and (iii) application of single-cell sequencing on tumor liquid biopsy can surveil and prevent the drug-resistant events during therapeutic treatment.^223^
